# Multi-omics analyses reveal altered gut microbial thiamine production in obesity

**DOI:** 10.3389/fmicb.2025.1516393

**Published:** 2025-06-17

**Authors:** Yu Xia, Longya Lu, Lulu Wang, Yanyan Qiu, Xingyin Liu, Weihong Ge

**Affiliations:** ^1^Department of Pharmacy, China Pharmaceutical University Nanjing Drum Tower Hospital, Nanjing, China; ^2^School of Basic Medicine and Clinical Pharmacy, China Pharmaceutical University, Nanjing, China; ^3^Department of Biochemistry, SUSTech Homeostatic Medicine Institute, School of Medicine, Southern University of Science and Technology, Shenzhen, Guangdong, China; ^4^Department of Pharmacy, Nanjing Drum Tower Hospital, The Affiliated Hospital of Nanjing University Medical School, Nanjing, China; ^5^Department of Pediatrics, The First Affiliated Hospital of Guangxi University of Chinese Medicine, Nanning, China

**Keywords:** microbiota, fecal metabolomics, thiamine, short chain fatty acids, obesity

## Abstract

**Objective:**

Accumulating evidence highlights the important role of B vitamins in maintaining the balance of gut microbial ecology and metabolism, however, few studies have focused on changes in B vitamins homeostasis in the gut and their associations with disease. This study aims to investigate the potential interplay between B vitamins, gut microbiota, and obesity.

**Methods:**

We conducted an integrated analysis of fecal shotgun metagenomics, fecal metabolome concerning B vitamins and short chain fatty acids (SCFAs), and obese phenotypes in a cohort of 63 participants, including 31 healthy controls and 32 individuals with obesity.

**Results:**

Metabolomic analysis identified significantly lower levels of fecal thiamine in individuals with obesity (*P*_Wilcoxon_ < 0.001). Fecal thiamine levels exhibited a positive correlation with HDL-C and a negative correlation with BMI, DBP, fasting serum insulin, HOMA-IR, triglycerides, and propionic acid. Binary logistics regression suggested that fecal thiamine deficiency may be a potential contributor to the onset of obesity (Odds ratio: 0.295). Metagenomic analysis indicated that the microbial composition in individuals with obesity was characterized by a predominance of potential opportunistic pathogens, a loss of complexity, and a decrease in thiamine-producing bacteria. Integrated analysis indicated that thiamine deficiency was positively associated with the depletion of thiamine auxotrophic bacteria in the obese microbiome. Functional analysis revealed that KOs content for enzymes involved in the microbial production of thiamine were significantly lower in obesity, including tRNA uracil 4-sulfurtransferase (ThiI, *P*_Wilcoxon_ = 0.001) and nucleoside-triphosphatase (NTPCR, *P*_Wilcoxon_ = 0.006), both of which were positively associated with fecal thiamine.

**Conclusion:**

Our study highlights the impairment of microbial thiamine production and its broad associations with gut microbiota dysbiosis and obesity-related phenotypes. Our findings provide a rationale for developing treatments that utilize thiamine to prevent obesity by modulating gut microbiota.

## 1 Introduction

Obesity is the excessive or abnormal accumulation of fat or adipose tissue in the body that impairs health via its association with the risk of development of diabetes mellitus, cardiovascular disease, hypertension, and hyperlipidemia (Vinnai et al., 2023). With its prevalence increasing dramatically over the past decades, obesity has become a global health epidemic that continues to worsen (Cani and Van Hul, 2024; Chanda and De, 2024; Deehan et al., 2024). In recent years, there has been a general increasing trend of overweight and obesity in China, and China has become the country with the largest number of overweight and obesity in the world (Wang et al., 2017; Hong et al., 2023; Wang et al., 2021). Obesity is a complex disease and has a multifactorial etiology. Generally, obesity is considered to be a multifactorial disease caused by a myriad of genetic and environmental factors (Xia et al., 2025).

Mounting evidence suggests that gut microbiota is a significant environmental factor contributing to the onset and progression of obesity and related metabolic disorders (Chanda and De, 2024; Lynch and Pedersen, 2016). Numerous cross-sectional studies have found that individuals with obesity exhibit alterations in gut microbiome composition and metabolism compared to healthy (lean) individuals (Ecklu-Mensah et al., 2023; Liu et al., 2017). Obesity is also referred to as chronic-low grade inflammation or “metabolic inflammation,” which is often the focus in the pathogenesis of several diseases such as coronary artery disease, atherosclerosis, and insulin resistance (Khanna et al., 2023). Perturbation in gut microbiota, changes in intestinal permeability, and increased level of bacterial lipopolysaccharide (LPS) have been identified as potential triggers of obesity-related low-grade inflammation (Portincasa et al., 2023). Recent clinical studies and animal models have provided evidence for a causal relationship between gut microbiota and obesity development through fecal microbiota transplantation experiments (Ecklu-Mensah et al., 2023; Liu et al., 2017). It is widely recognized that modulating gut microbiota through targeted approaches might hold great promise for treating overweight and obesity (Cani and Van Hul, 2024).

B vitamins refer to a group of water-soluble organic compounds, including vitamin B1 (thiamine), B2 (riboflavin), B3 (niacin), B5 (pantothenic acid), B6 (pyridoxine), B7 (biotin), B9 (folate), and B12 (cobalamin) (Said and Nexo, 2018). They are essential micronutrients for the normal growth and development of microbes as well as the mammalian host (Hossain et al., 2022). Human gut commensals have been known to be significant producers of B vitamins, which serve as an essential cofactor/coenzyme involved in microbial metabolism (Das et al., 2019). For example, thiamine is involved in functions of multiple enzymes necessary for the metabolism of carbohydrates, fatty acids and amino acids (National Institute of Diabetes and Digestive and Kidney Diseases, 2012; Rudzki et al., 2021), contributing to the microbial production of short chain fatty acids (SCFAs) in the gut (Takeuchi et al., 2023; Wang et al., 2022). Microbial species that can synthesize vitamins *de novo* are known as vitamin prototrophs, while other microbial species that lack biosynthetic pathways and rely on exogenous sources are known as vitamin auxotrophs (Das et al., 2019; Rudzki et al., 2021). Most microbe-mediated vitamin production takes place in the large intestine and hereby produced vitamins can feeding the auxotrophic species or be absorbed by the host (Das et al., 2019). Emerging evidence indicates that gut B vitamins play crucial roles in maintaining gut homeostasis (Uebanso et al., 2020; Wan et al., 2022; Zhan et al., 2022). They support the survival and fitness of specific bacteria, regulate intestinal immunity, and suppress the colonization of pathogenic bacteria (Uebanso et al., 2020; Kunisawa and Kiyono, 2013). Particularly, the cross-feeding of B vitamins between prototrophic and auxotrophic species is expected to significantly contribute to the balance of microbial communities in the distal gut (Rodionov et al., 2019).

In individual with obesity, the functional potential of gut microbiota related to metabolism of cofactors and vitamins has been reported to change (Liu et al., 2017; Gao et al., 2018; Belda et al., 2022). Despite the well-established role of gut bacteria in vitamin production, and the understanding that impaired bacterial production of B vitamins can affect both microbial communities and host metabolism and inflammation (Belda et al., 2022), few studies have attempted to quantify B vitamins content in the human gut or explore the interplay between B vitamins, gut microbiota, and disease phenotypes. Recently, for the first time, we developed a rapid, robust, and reliable method for the simultaneous analysis of B vitamins in human feces using HPLC-ESI-MS/MS, revealing lower levels of fecal thiamine and niacin in individuals with obesity compared with healthy individuals (Xia et al., 2024). In this study, we further employed shotgun metagenomics to capture alterations in gut microbiota within a well-characterized cohort. We also assessed microbiota-derived metabolites—SCFAs. Based on the integrated analysis of clinical information and multi-omics data, our study provides insights into the role of thiamine in the microbiota dysbiosis and its association with obesity.

## 2 Materials and methods

### 2.1 Subject recruitment

The study participants were recruited from The Nanjing Drum Tower Hospital, the Affiliated Hospital of Nanjing University Medical School. The inclusion criteria for healthy controls and patients are as follows: (i) Han Chinese; (ii) Registered residence in Nanjing and its adjacent areas; (iii) diagnosis of simple obesity (BMI ≥ 30 kg/m^2^) or normal weight (18.5 ≤ BMI < 25 kg/m^2^) according to the World Health Organization (WHO) criteria (Wit et al., 2023). The exclusion criteria for obesity are as follows: (i) secondary causes of obesity; (ii) Other acute and chronic diseases besides obesity and related complications; (iii) antibiotic treatment within 2 months before sample collection; (iv) history of drug use within 1 month, including traditional Chinese medicine; (v) history of tobacco or alcohol abuse; (vi) undergoing weight loss treatment; and (vii) intake of probiotics, vitamin supplements, and other healthcare products that may contain B vitamins within 1 month. The exclusion criteria for healthy controls are as follows: (i) Abnormal physical examination result or self-reported physical discomfort; (ii) history of medication within 2 months prior to sample collection; (iii) history of tobacco or alcohol abuse; and (iv) intake of probiotics, vitamin supplements and other health care products that may contain B vitamins within 1 month. All subjects gave their written or verbal informed consent for inclusion before they participated in the study. This study was approved by the Ethics Committees of Nanjing Drum Tower Hospital, the Affiliated Hospital of Nanjing University Medical School (registration number: 2023–343), and was performed in accordance with the principle of the Helsinki Declaration II.

### 2.2 Fecal and serum sample collection

Participants were instructed to fast overnight before their visits. Blood samples were collected in the morning during their hospital appointments, centrifuged within 1 h and then stored at −80°C until the sample preparation and analysis. Fecal samples were collected using sterile sampling devices at the hospital or at home. The fecal samples were delivered to the lab at low temperatures within 1 h. Upon receipt, the researchers aseptically divided each sample into three aliquots, which were immediately stored at −80°C until analysis.

### 2.3 Fecal DNA extraction

Total microbial genomic DNA samples were extracted using the Mag-Bind Soil DNA Kit (M5635-02) (Omega Bio-Tek, Norcross, GA, USA), following the manufacturer's instructions, and stored at −80°C prior to further assessment. The quantity and quality of extracted DNAs were measured using a Qubit™ 4 Fluorometer, and agarose gel electrophoresis, respectively.

### 2.4 Shotgun metagenomic sequencing, quality control, and microbial taxonomic profiling

The extracted microbial DNA was processed to construct metagenome shotgun sequencing libraries with insert sizes of 400 bp by using Illumina TruSeq Nano DNA LT Library Preparation Kit. Each library was sequenced by Illumina NovaSeq platform (Illumina, USA). Then, the raw sequencing reads were processed to obtain quality-filtered reads without human host DNA for further analysis (Chen et al., 2018). First, sequencing adapters were removed from sequencing reads using Cutadapt (v1.2.1). Secondly, low quality reads were trimmed using a sliding-window algorithm in fastp. Thirdly, reads were aligned to the host genome of human using BMTagger, and the reads that matched the host were discarded to remove host contamination (Rotmistrovsky and Richa, 2011). Once quality-filtered reads were obtained, taxonomical classifications of metagenomics sequencing reads from each sample were performed using Kraken2 against an RefSeq-derived database (Wood et al., 2019). Metagenome sequencing data can be accessed on the SRA database, accession number: PRJNA1110638.

### 2.5 Functional annotation of metagenomic data

The high-quality reads were then assembled to generate contigs (longer than 300bp) using Megahit (v1.1.2) (Li et al., 2015). The non-redundant contigs was obtained by aligning them against the NCBI-nt database by mmseqs2 setting with a threshold of more than 95% identity over 90% of the length (Steinegger and Soding, 2017). MetaGeneMark was used to predict the genes in the contigs (Zhu et al., 2010). The functionality of the non-redundant genes were obtained by annotated using mmseqs2 with the “search” mode against the protein databases of KEGG, MetaCyc and VFDB databases (Steinegger and Soding, 2017), respectively. Kyoto Encyclopedia of Genes and Genomes (KEGG) orthologs (KOs) were obtained using KOBAS (Bu et al., 2021).

### 2.6 Anthropometric and biochemical measurements

The height and weight of all participants were measured in light clothing without shoes, and then the body mass index (BMI) was calculated as weight (kg) divided by height squared (m^2^). Blood pressure was measured at resting state following the standard operating procedures. Fasting blood glucose, serum alanine aminotransferase (ALT), aspartate aminotransferase (AST), uric acid, triglycerides, total cholesterol, high-density lipoprotein cholesterol (HDL-C), and low-density lipoprotein cholesterol (LDL-C) were measured by the Beckman Coulter clinical chemistry system (Pasadena, California, USA). Fasting insulin was measured by a chemiluminescence immunoassay (Cobas e801, Roche). Insulin resistance index (HOMA-IR) was calculated using homeostasis model assessment methods, defined as fasting insulin × fasting glucose/22.5. Serum lipopolysaccharide was measured using a human ELISA kit according to the manufacturer's instructions (EIAab, Wuhan, China).

### 2.7 Fecal B vitamins metabolomics

Fecal B vitamins were measured by high-performance liquid chromatography-electrospray ionization-tandem mass spectrometry (HPLC-ESI-MS/MS) as we previously established (Xia et al., 2024), including vitamins B1, B2, B3, B5, B6, and B7. Fecal vitamin B9 and B12 in fecal supernatant was quantified via chemiluminescent immunoassay (Atellica IM Analyzer; Siemens Healthcare, Ballerup, Denmark).

### 2.8 Fecal SCFAs metabolomics

Fecal SCFAs were measured by gas chromatography-mass spectrometry (GC-MS) as previously described with minor modifications (Han et al., 2018), including acetic acid, propionic acid, isobutyric acid, butyric acid, isovaleric acid, valeric acid, and caproic acid.

### 2.9 Co-occurrence network analysis

The differential species were clustered according to Spearman's correlation between their relative abundances in all samples regardless of case-control status, and the co-occurrence network was visualized with Cytoscape software if the |Spearman's rank correlation coefficient| > 0.4.

### 2.10 Statistical analysis

Descriptive statistics for continuous variables are reported as mean ± standard deviation if normally distributed or as the median (P25, P75) if they are not. Categorical variables are presented as counts. The concentrations of SCFAs and B vitamins in feces were log-transformed (using natural logarithm) prior to analysis. Differences between two groups were assessed using two-sided Wilcoxon rank-sum tests. Differentially abundant taxa or functional modules were identified using linear discriminant analysis (LDA) through the Linear Discriminant Analysis Effect Size (LEfSe) method. The associations between differential metabolites, differential microbiota, and obesity phenotypes were analyzed using Spearman correlation analysis. The *P* values are adjusted using the Benjamini–Hochberg method to control the false discovery rate (FDR) in multiple testing scenarios. Box plots were created, and statistical analyses of phenotypic data were performed using Prism 9 (GraphPad Software, CA). Binary logistic regression was performed using SPSS (IBM SPSS 26.0, SPSS Inc.). Statistical analyses and graphs involved in microbial data were done with R version v4.2.1.

## 3 Results

### 3.1 Basic characteristics of the recruited subjects

In the present study, a total of 31 healthy controls and 32 patients with obesity were recruited in accordance with the inclusion and exclusion criteria strictly. The demographic index and clinical characteristics of the study population were summarized. As shown in Supplementary Table S1, there were no significant differences in gender, age, and ethnicity between obese individuals and healthy individuals. Compared with the obese group, serum HDL-C was significantly higher in the healthy group. In contrast, SBP, DBP, fasting blood glucose, HOMA-IR, ALT, AST, triglycerides, total cholesterol, and LDL-C were significantly higher in obese individuals as compared with healthy controls. These results indicated dysfunction of glucose and lipid metabolism in obese patients.

### 3.2 Fecal thiamine levels were associated with obesity-related phenotypes

**Figure 1:**
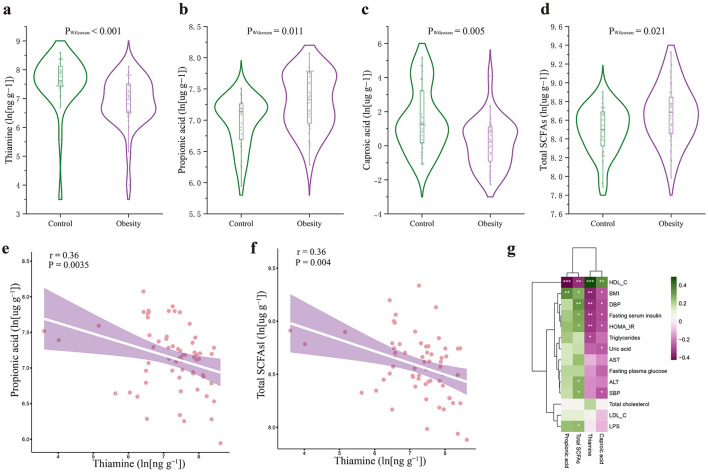
Alterations of fecal metabolites in obesity and their association with obesity-related phenotypes. **(a–d)** The comparison of the fecal thiamine levels, fecal propionic acid levels, fecal caproic acid levels, and fecal total SCFAs levels in control and obese individuals, respectively. **(e)** The negative correlation between fecal thiamine and fecal propionic acid. **(f)** The negative correlation between thiamine and total SCFAs. **(g)** Heat map of the Spearman's rank correlation coefficient of clinical phenotypes and differential fecal metabolites. Green color represents a positive correlation while purple color represents a negative correlation. HDL-C, high-density lipoprotein cholesterol; LDL-C, low-density lipoprotein cholesterol; LPS, lipopolysaccharide; SBP, systolic blood pressure; DBP, diastolic blood pressure; HOMA-IR, insulin resistance index; ALT, alanine transaminase; AST, aspartate transaminase; BMI, body mass index; SCFAs, short chain fatty acids. ^*^p < 0.05, ^**^p < 0.01, ^***^p < 0.001.

We used HPLC-ESI-MS/MS and chemiluminescence immunoassay to detect eight B vitamins levels in fecal samples. In univariate analyses, we found that patients with obesity showed a significant reduction of fecal thiamine levels (Supplementary Table S2, Figure 1a), while the levels of the other seven B vitamins showed no significant differences, including vitamin B2, B3, B5, B6, B7, B9, and B12 (Supplementary Table S2). We next examined whether fecal levels of thiamine distinguished between patients with obesity and controls. We found that lower thiamine level was associated with a higher risk of obesity (odds ratio 0.295, 95% confidence interval [CI] 0.126–0.693; *p* < 0.001). In addition, correlation analysis revealed that fecal thiamine level was highly correlated with obesity-related phenotypes. As shown in Figure 1g, fecal thiamine was positively correlated with HDL-C and negatively correlated with BMI, DBP, fasting serum insulin, HOMA-IR, triglycerides. These results highlighted the close associations between fecal thiamine and obesity-related phenotypes.

### 3.3 Fecal thiamine levels were associated with fecal SCFAs levels

Given that thiamine is mainly involved in the metabolism of carbohydrates, we also measured fecal SCFAs, the primarily products of microbial carbohydrate metabolism. In univariate analyses, we found that patients with obesity had significantly higher fecal levels of caproic acid, propionic acid, and total SCFAs than healthy controls (Supplementary Table S3, Figures 1b–d). We next examined whether fecal levels of individual SCFA types distinguished between patients with obesity and controls. We found that higher fecal levels of propionic acid (odds ratio 6.73, 95% CI 1.655–27.362; *p* = 0.011), and total SCFAs (odds ratio 9.022, 95% CI 1.203–67.66; *p* = 0.021) were associated with a higher risk of obesity. In contrast, higher fecal levels of caproic acid (odds ratio 0.597, 95% CI 0.410–0.868; *p* = 0.005) was associated with a lower risk of obesity. In addition, as shown in Figure 1g, fecal propionic acid was negatively correlated with HDL-C and positively correlated with BMI; fecal total SCFAs was negatively correlated with HDL-C, while positively correlated with BMI, DBP, SBP, fasting blood glucose, insulin resistance index, ALT, and LPS.

Next, we performed correlation analysis to explore the associations between fecal thiamine and fecal SCFAs. As shown in Figures 1e, f, we found that fecal thiamine was negatively associated with propionic acid and total SCFAs.

### 3.4 Taxonomic alternation of gut microbiota in obesity

**Figure 2:**
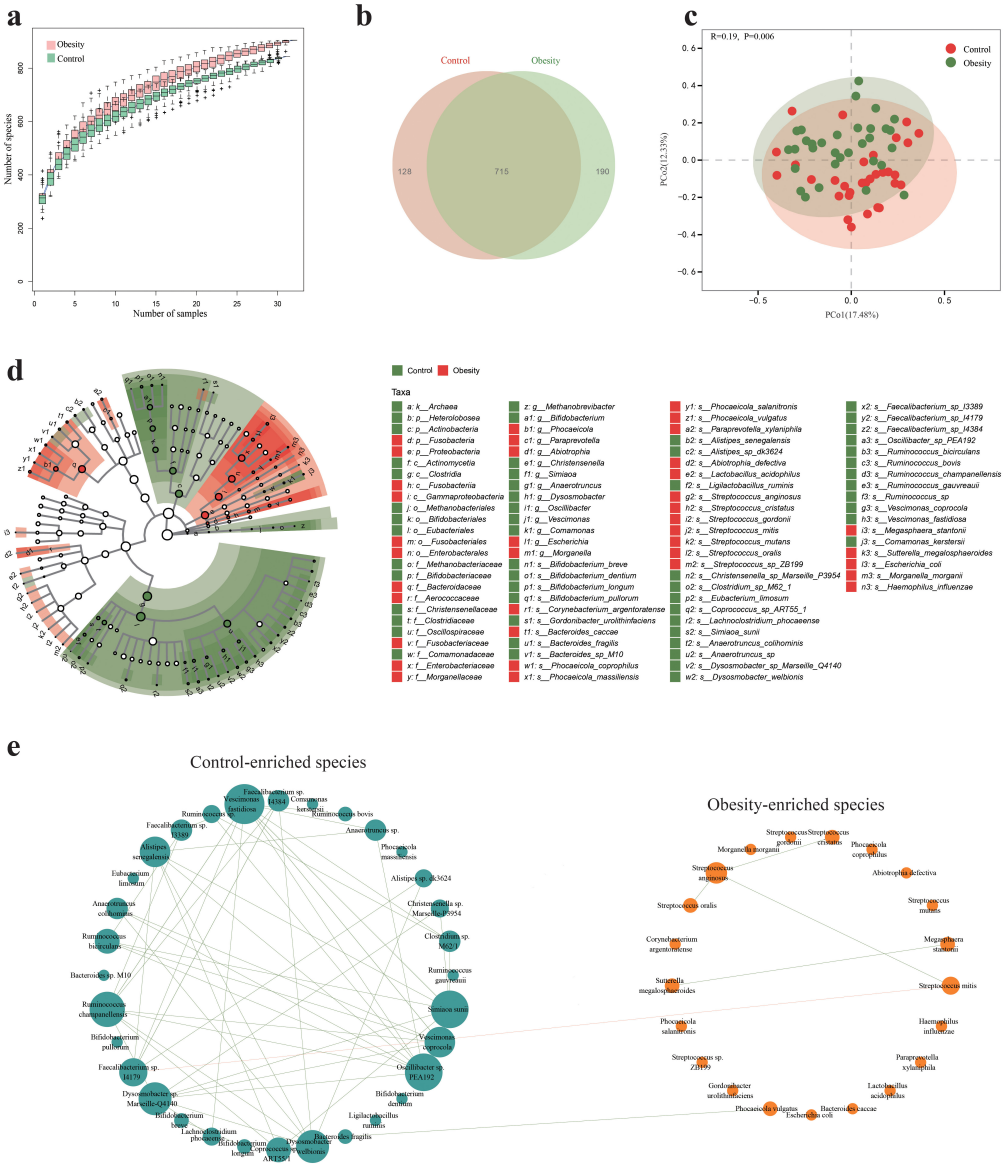
Alterations of gut microbiota in obesity. **(a)** Rarefaction curves generated from the identified species. **(b)** Venn diagram of the observed species in the fecal microbiota of obese patients and healthy controls. **(c)** Principal Coordinate analysis (PCoA) of the microbiota based on the Bray-Curtis for obese patients and healthy controls. **(d)** Cladogram generated by LEfSe indicating the phylogenetic relatedness of the discriminant taxa. Red color indicate taxa were enrichment in obesity, green color indicate taxa were enrichment in control. **(e)** Co-occurrence network deduced from 53 species enriched in obese subjects and healthy controls. Sizes of the nodes represent the degree of connectivity. Green edges, Spearman's rank correlation coefficient > 0.4, P_FDR_ < 0.05; red edges, Spearman's rank correlation coefficient < −0.4, P_FDR_ < 0.05.

As gut microbiota is the primarily source of vitamins in the distal gut, we analyzed the gut microbiota using metagenome shotgun sequencing across the 63 fecal samples. In total, we obtained millions of high-quality reads from the fecal samples. These reads were annotated into 1,033 microbial species (Figure 2b). Rarefaction curves generated from the species suggested that high sampling coverage was achieved in all samples (Figure 2a). This indicated that the sequencing depth was sufficient for the investigation of the fecal microbiota. Venn diagram displayed 715 species were shared between the obese groups and healthy control groups, while 190 and 128 species were unique to obesity and healthy control, respectively (Figure 2b). The five most abundant phyla and 20 most abundant genera and 20 most abundant species in both groups are shown in Supplementary Figures S1a–c.

We then compared the alpha diversity between the two groups, measured by the Simpson, chao1, ace, and Shannon indexes. No significant differences were found for these indexes between the two groups (Supplementary Figures S2a–d), indicating the alpha diversity of the gut microbiota was similar in obese and healthy groups. To further explore whether the microbial composition of obese subjects was different from that of healthy controls, beta diversity analysis was performed using Principal Coordinates Analysis (PCoA) based on Bray-Curtis. This analysis indicated that the overall microbiota composition of the obese group was significantly different from that of the control group (Figure 2c).

To identify the key gut microbes that most likely account for differences between obese patients and healthy controls, LEfSe analysis was conducted. As shown in Figure 2d and Supplementary Figure S3, multiple taxonomic differences between patients with obesity and healthy control subjects were identified (LDA score > 2). Notably, the control-enriched species were dominated by potential beneficial bacteria, such as *Bifidobacterium* spp., *Faecalibacterium* spp., *Dysosmobacter welbionis etc*. However, the obesity-enriched species were dominated by potential pro-inflammatory or pathogenic microbiota, such as *Streptococcus* spp., *Escherichia coli, Morganella morganii, Haemophilus influenzae* etc. Additionally, the control-enriched species were characterized by well-documented thiamine-producing bacteria (e.g., *Bifidobacterium* spp.) and thiamine-consuming bacteria (e.g., *Alistipes* spp., *Ruminococcus* spp.).

Subsequently, we constructed a species network depicting the correlation between obesity-associated gut microbial markers. As shown in Figure 2e and Supplementary Table S4, control-enriched species were more highly interconnected than obesity-enriched species. Notably, several thiamine auxotrophic bacteria, such as *Faecalibacterium* sp. I4179, *Ruminococcus* spp., and *Alistipes* spp., exhibited relatively more connections with other bacteria.

### 3.5 Microbiota alterations were associated with obesity-related phenotypes

**Figure 3:**
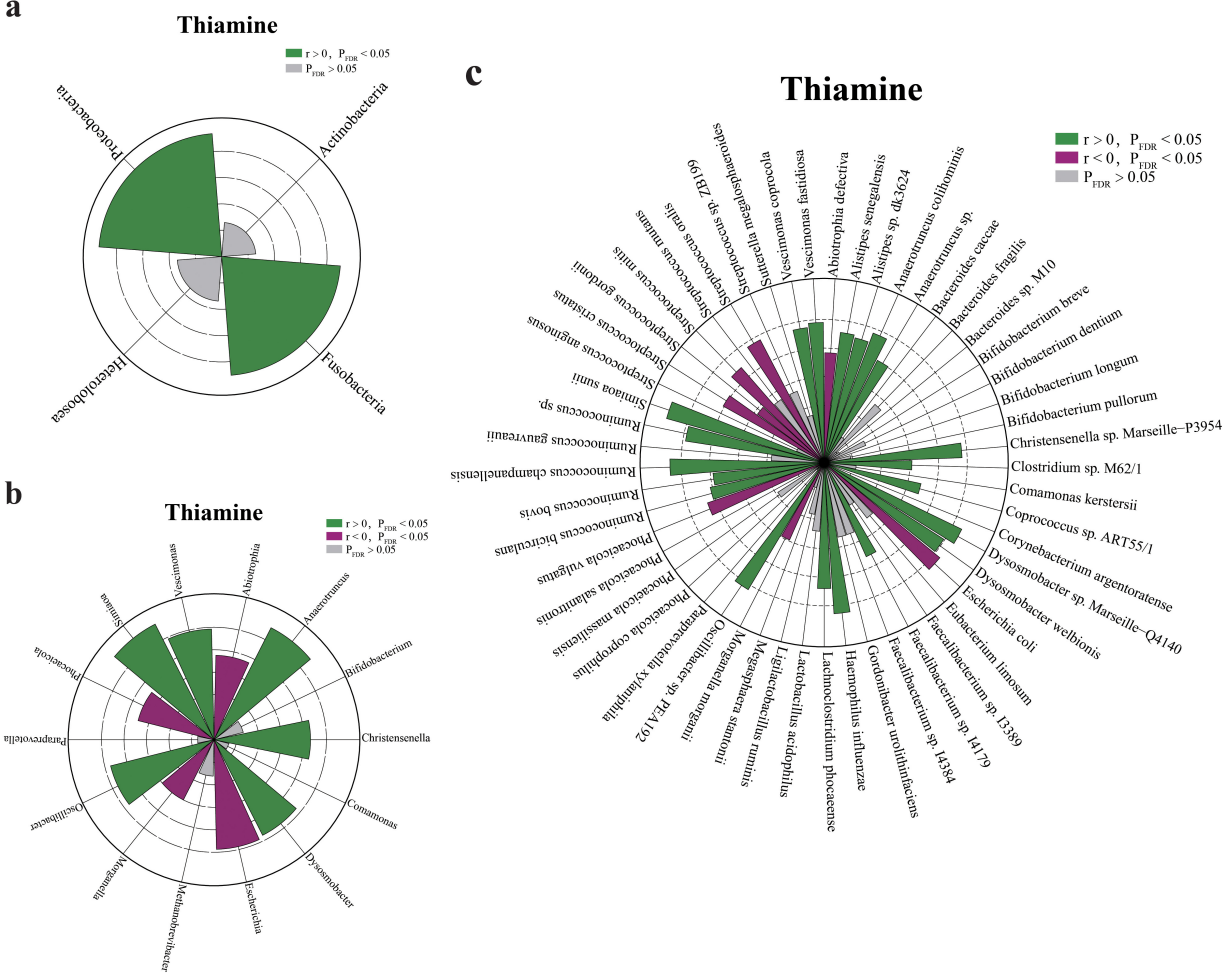
Associations of gut microbial taxa with fecal thiamine. **(a–c)** The effects of phylum, genus, and species associated with thiamine, respectively. Green sectors indicate significant positive associations, purple sectors indicate significant negative associations and gray sectors indicate insignificant associations. Dashed circles indicate the scale of correlation coefficient r from 0.1 to 0.6.

To further explore the potential correlations of host phenotypic changes with differential microbial species in obesity, correlation analysis was performed. Extensive associations were observed between the microbial markers and clinical indexes (Supplementary Figure S4, Supplementary Table S5). Next, we established microbial associations with fecal thiamine. At the phylum level, we noted that fecal thiamine was negatively associated with *Fusobacteria* and *Proteobacteria* (Figure 3a, Supplementary Table S6). At the genus level, we noted that fecal thiamine was positively associated with *Vescimonas, Oscillibacter, Dysosmobacter, Christensenella, Anaerotruncus*, and *Simiaoa*, while negatively associated with *Abiotrophia, Escherichia, Phocaeicola*, and *Morganella* (Figure 3b, Supplementary Table S6). At the species level, thiamine was observed to be positively correlated with several anti-inflammatory or anti-obesity bacteria (e.g., *Ruminococcus bicirculans, Dysosmobacter welbionis*, etc.) and negatively correlated with multiple potential pathogenic bacteria (e.g., *Escherichia coli, Haemophilus influenzae*, etc.) (Figure 3c, Supplementary Table S6). Additionally, we observed broad positive associations between thiamine and thiamine auxotrophic bacteria, such as *Faecalibacterium* sp. I4179, *Ruminococcus* sp, *Ruminococcus bicirculans*, etc.

### 3.6 Low fecal thiamine levels were associated with impaired thiamine biosynthesis by gut microbiota

**Figure 4:**
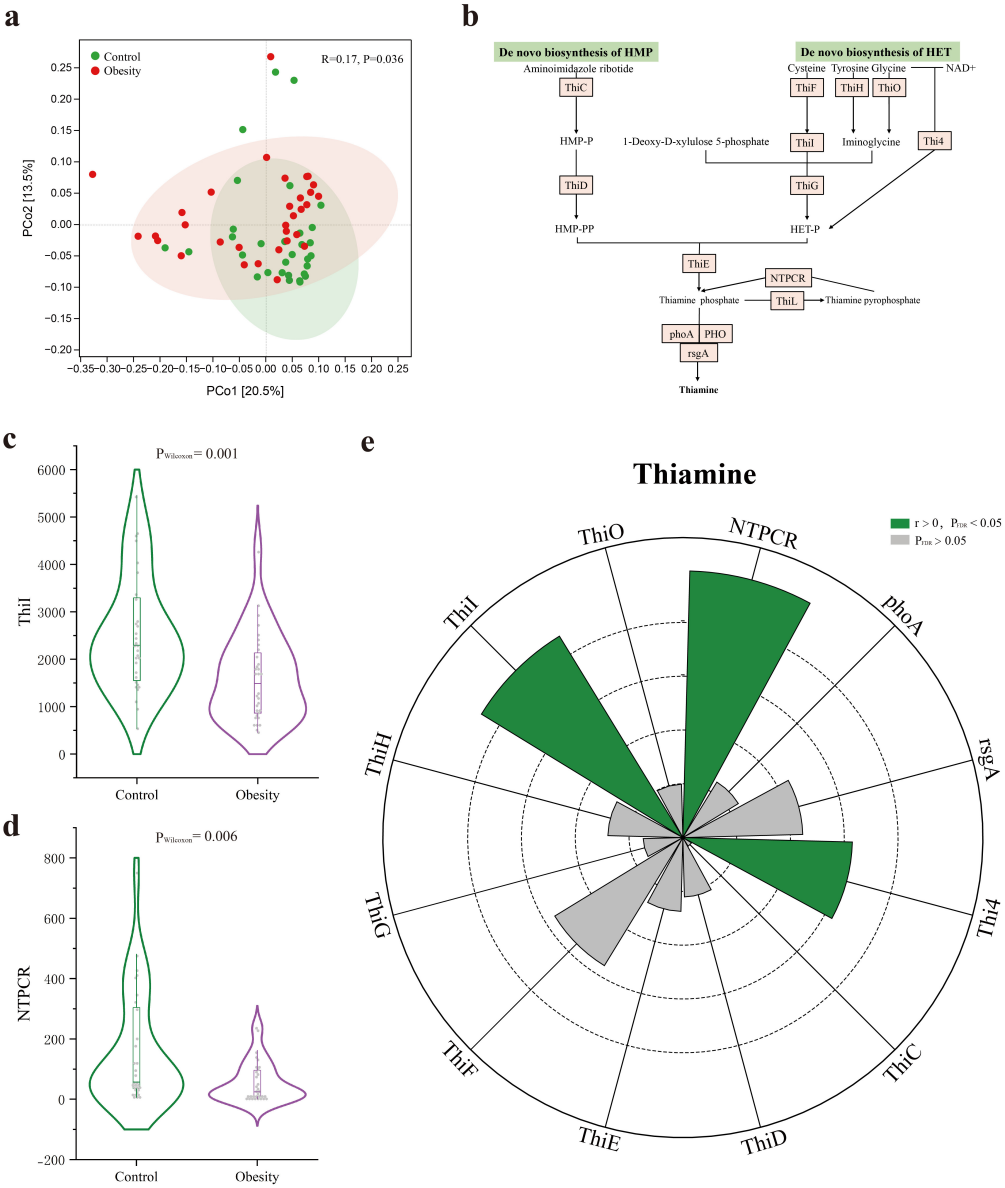
The effect of Kyoto Encyclopedia of Genes and Genomes orthologs (KOs) on thiamine. **(a)** Principal Coordinate analysis (PCoA) of the KOs based on the Bray-Curtis for obese patients and healthy controls. **(b)** Schematic showing the microbial pathway of thiamine biosynthesis *de novo*. Enzymes are shown by orange boxes. **(c)** The comparison of the ThiI count in control and obese individuals. **(d)** The comparison of the NTPCR count in control and obese individuals. **(e)** The effects of 12 enzymes associated with thiamine production. Green sectors indicate significant positive associations and gray sectors indicate insignificant associations. Dashed circles indicate the scale of correlation coefficient r from 0.1 to 0.5. ThiI, tRNA uracil 4-sulfurtransferase; ThiO, glycine oxidase; NTPCR, nucleoside-triphosphatase; phoA, alkaline phosphatase; rsgA, ribosome biogenesis GTPase; THI4, cysteine-dependent adenosine diphosphate thiazole synthase; THIC, phosphomethylpyrimidine synthase; THID, hydroxymethylpyrimidine/phosphomethylpyrimidine kinase; THIE, thiamine-phosphate pyrophosphorylase; THIF, sulfur carrier protein ThiS adenylyltransferase THIG, thiazole synthase; THIH, 2-iminoacetate synthase.

To evaluate the functional alteration of gut microbiota in obesity, functional metagenomic analysis was carried out. We identified 6,556 KOs. PCoA analysis based on KOs revealed differences in microbial functions between obesity and healthy control (Figure 4a). We next investigated the KOs content for enzymes involved in the microbial production of thiamine (Figure 4b). We found that two KOs—tRNA uracil 4-sulfurtransferase (ThiI) and nucleoside-triphosphatase (NTPCR)—were significantly higher in healthy controls (Figure 4c). Correlation analysis revealed that fecal thiamine was significantly positive correlated with ThiI, NTPCR, and cysteine-dependent adenosine diphosphate thiazole synthase (Thi4) (Figure 4d, Supplementary Table S7).

Additionally, we performed correlation analysis to explore the associations between enzymes involved in the microbial production of thiamine and obesity-related phenotypes. Specifically, we noted that ThiI was positively associated with HDL-C, while negatively associated with fasting serum insulin (Supplementary Figure S5, Supplementary Table S8).

### 3.7 Functional alternation of gut microbiota in obesity

**Figure 5:**
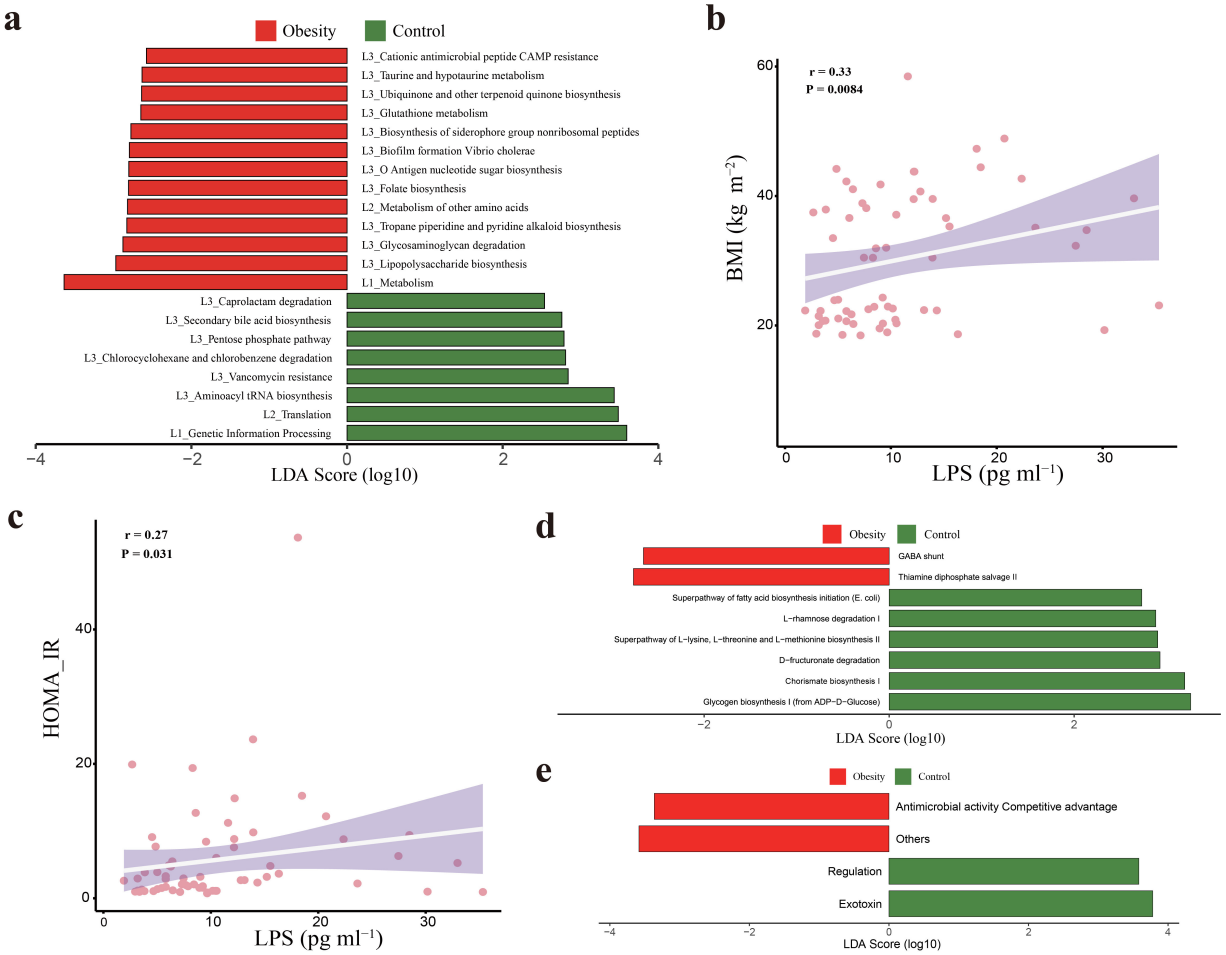
Functional characterization of the obese microbiome and corresponding metabolic changes. **(a)** Differential KEGG pathways on level 1, level 2, and level 3, identified by LEfSe from the control and obese groups. **(b)** The positive correlation between LPS and HOMA-IR. **(c)** The positive correlation between LPS and body mass index (BMI). **(d, e)** Differential MetaCyc pathways, and VFDB categories identified by LEfSe from the control and obese groups. Cutoffs for LEfSe analysis were LDA score [log 10] > 2. Red bars indicate function were enrichment in obesity, and green bars indicate function were enrichment in healthy controls. KEGG, Kyoto Encyclopedia of Genes and Genomes; LPS, lipopolysaccharide; HOMA-IR, insulin resistance index; VFDB, virulence Factors of Pathogenic Bacteria; LEfSe, Linear discriminant analysis Effect Size; LDA, linear discriminant analysis.

Next, we performed a functional enrichment analysis. LEfSe analysis revealed that 21 KEGG pathway on level 1, level 2, and level 3 significantly differed between obesity and healthy controls (Figure 5a). Notably, it was observed that multiple pathways related to microbial invasiveness and virulence, including “glycosaminoglycan degradation,” “antigen nucleotide sugar biosynthesis,” “lipopolysaccharide biosynthesis,” “cationic antimicrobial peptide CAMP resistance pathways,” and “biofilm formation Vibrio cholerae” were significantly enriched in the obese group. Consistently, we found a significant increase of serum LPS in obese group (Supplementary Table S1). Furthermore, correlation analysis revealed that serum LPS levels are positively correlated with BMI and HOMA-IR (Figures 5b, c).

Microbial metabolic pathways were also annotated using the MetaCyc database. LEfSe analysis revealed that eight MetaCyc pathways were significantly altered in subjects with obesity. Particularly, we noted that MetaCyc pathway “thiamine diphosphate salvage II” was significantly enriched in the obese group compared with the healthy controls (Figure 5d).

We also annotated the virulence genes based on the virulence factor database (VFDB), and the virulent factors of obese group and healthy controls were further classified into categories. LEfSe analysis revealed that four categories were significantly altered in subjects with obesity. Particularly, we noted that an enrichment of “antimicrobial activity/competitive advantage” in obesity (Figure 5e).

## 4 Discussion

B vitamins, which serve as precursors for essential cofactors and coenzymes, are crucial micronutrients needed in small quantities for the proper growth and development of organisms (Hossain et al., 2022; Wan et al., 2022). Due to the efficient absorption of dietary vitamins in the small intestine, they rarely reach the large intestine, where harbors the highest density of microbes in the human gut (Wan et al., 2022; Zhan et al., 2022; Steinert et al., 2020). It is well-established that gut microbiota has the capacity to synthesize B vitamins (Wan et al., 2022; Yang et al., 2024). In this study, we found that individuals with obesity had significantly lower fecal thiamine levels compared to healthy controls. An integrated analysis of the gut metabolome, microbiome and obesity-related phenotypes indicated that low fecal thiamine levels were associated with impaired gut microbial thiamine biosynthesis, an imbalanced gut microbiota, and obesity-related phenotypes.

In the present study, we found that fecal thiamine was negatively correlated with HOMA-IR, triglycerides and DBP, and positively correlated with HDL-C. Previous research on rodents has shown that high-dose thiamine can counter dyslipidemia (Babaei-Jadidi et al., 2004) and fatty liver (Kalyesubula et al., 2021), and have potential in preventing obesity and metabolic disorders (Tanaka et al., 2010). Additionally, the impact of thiamine on cardiometabolic health has been well documented, as confirmed in a randomized controlled trial demonstrating that thiamine can reduce DBP (Alaei-Shahmiri et al., 2015). It is worth mentioning that the recommended daily dose of vitamin is difficult to reach the distal gut. However, when administered in large amounts beyond the threshold of small intestinal absorption, a portion may escape absorption and directly modulate gut microbiota (Steinert et al., 2020). These findings suggest that high-dose thiamine might be a potential therapeutic candidate for the treatment of obesity by modulating gut microbiota.

Consistent with other studies (Chanda and De, 2024; Pinart et al., 2021), we observed a dysbiotic gut microbiota in individuals with obesity. PCoA analysis indicated significant differences in bacterial communities between the two groups. Intriguingly, we found extensive associations between fecal thiamine and microbial species. Specifically, fecal thiamine was positively associated with *Faecalibacterium* sp I4179, *Alistipes* species (*Alistipes senegalensis* and *Alistipes* sp dk3624), and four of the five *Ruminococcus* species (*Ruminococcus* sp, *Ruminococcus champanellensis, Ruminococcus bovis*, and *Ruminococcus bicirculans*). It has been reported that thiamine deficiency and low thiamine environment were detrimental to *Alstipes* and *Faecalibacterium prausnitzii* (Zhan et al., 2022). Additionally, *Ruminococcaceae* lack the thiamine synthesis pathway and required a supply of thiamine from external source for their growth (Park et al., 2022). Furthermore, we observed that healthy controls were enriched with well-documented thiamine-producing microbial taxa [e.g., *Actinobacteria* (Wan et al., 2022), *Bifidobacterium* (Nicholson et al., 2012), and *Bacteroides fragilis* (Wan et al., 2022)], and thiamine-consuming microbial taxa [e.g., *Ruminococcaceae* (Park et al., 2022), *Alstipes* spp. (Zhan et al., 2022), and *Faecalibacterium* spp. (Zhan et al., 2022)] compared to those with obesity.

The gut microbiota is composed of a vast community of microbes. These diverse bacterial species hosted within the human gut form a complex ecological interaction web, interacting with one another either positively or negatively to create and adapt to suitable living conditions (Wang et al., 2024). It has been reported that deficiencies in micronutrient (e.g., vitamin) can significantly impact the composition and function of gut microbiota (Hibberd et al., 2017). Notably, we found that the control-enriched species exhibited substantially more connections than those enriched in obesity, indicating that the complexity of gut microbiota is disrupted in obesity. Specifically, we observed that certain thiamine auxotrophic bacteria (e.g., *Ruminococcus champanellensis, Alistipes senegalensis*, and *Faecalibacterium sp*. I4179) exhibited relatively more connections with others compared to thiamine prototrophic bacteria (e.g., *Bifidobacterium dentium, Bifidobacterium longum*, and *Bacteroides fragilis*). A recent study indicated that low species richness was associated with a lack of metabolites available for consumption, favoring species that are self-sufficient in producing these metabolites, such as phosphate, glucose, galactose and choline (Marcelino et al., 2023). Accordingly, a plausible explanation for our findings is that a thiamine-sufficient environment in healthy controls favors the growth of thiamine auxotrophic bacteria, thus contributing to the diversity and complexity of the microbiota. These findings support the notion that B vitamins deficiency can lead to the loss of vitamin auxotrophic species, the destruction of complexity of microbial community, and trigger the competitive state in the intestinal ecosystem (Zhan et al., 2022; Marcelino et al., 2023).

The gut microbiota has a remarkable ability to synthesize and utilize B vitamins (Yang et al., 2024). In this study, we observed a higher content of ThiI in healthy controls, which is involved in the *de novo* biosynthesis of thiamine through microbial pathways. Correlation analysis further revealed a significant positive correlation between fecal thiamine and the enzymes ThiI and Thi4. Consistently, we observe an enrichment of species possessing genes encoding ThiI in healthy controls, such as *Dysosmobacter welbionis, Lachnoclostridium phocaeense, Vescimonas fastidiosa* etc. These species also exhibited positive associations with fecal thiamine levels. Additionally, we observed a higher content of NTPCR in healthy control. NTPCR is one of key enzyme involved in the conversion of thiamine pyrophosphate (TPP) to thiamine. Correlation analysis showed a positive association between fecal thiamine and NTPCR. Taken together, these findings indicated that the deficiency of fecal thiamine in obesity was associated with the impaired capability of thiamine production by gut microbiota.

Additionally, MetaCyc pathway enrichment analysis revealed that “thiamine pyrophosphate salvage II” was enriched in the microbiome of obese individuals compared to healthy controls. This pathway described the biosynthesis of TPP through the salvage of two thiamine fragments (i.e., HET and HMP) from the environment (Du et al., 2011; Culp and Goodman, 2023). One plausible explanation for this finding is that obese microbiome with a reduced ability of thiamine biosynthesis *de novo*, may rely on utilizing thiamine fragments in an environment lacking thiamine, which is more efficient and energy-saving (Manzetti et al., 2014). It has been reported that gut opportunistic pathogens can adjust their metabolism for an advantage in nutrient competition by recognizing signals and nutrients (Cameron and Sperandio, 2015). Notably, a strong negative correlation was observed between fecal thiamine levels and the abundance of *Escherichia coli*, which has been well demonstrated possess the MetaCyc pathway “thiamine diphosphate salvage II” (Culp and Goodman, 2023).

SCFAs are generated by gut microbial fermentation of mainly undigested dietary carbohydrates, specifically resistant starch and dietary fiber and, to a lesser extent, dietary and endogenous proteins (Wang et al., 2022; Liu et al., 2018; Krautkramer et al., 2021). Notably, thiamine plays a crucial role in the metabolism of amino acids and carbohydrates and is involved in energy production reactions (National Institute of Diabetes and Digestive and Kidney Diseases, 2012), contributing to the production of SCFAs by gut microbiota (Wang et al., 2022). Thiamine serves as an indispensable cofactor for numerous enzymes involved in the production of SCFAs, including pyruvate dehydrogenase and α-ketoglutarate dehydrogenase (Yoshii et al., 2019; Kerns et al., 2015). In this study, we observed significantly higher levels of propionic acid and SCFAs in the obese group. It has been reported that, in addition to the dietary content, thiamine status in serum is also dependent on the dietary carbohydrate status since this vitamin is involved in their metabolism, and it was demonstrated that an increase of dietary carbohydrate intake results with a decrease in plasma and urine levels thiamine (Rudzki et al., 2021). The obese patients in our study generally reported a preference for carbohydrate-rich foods, it is reasonable to speculate that the observed deficiency of fecal thiamine may be partly due to its excessive utilization by microbes for SCFA production, particularly propionic acid.

Additionally, we observed a significant increase of *Escherichia coli* in individuals with obesity. Notably, overflow metabolism, a phenomenon that refers to metabolic processes prioritizing growth rate over efficiency in specific nutrient environments (Basan et al., 2015), has been largely studied in *Escherichia coli* (Culp and Goodman, 2023). During overflow metabolism, gut microbes under conditions of high resource availability grow faster by utilizing fermentation rather than aerobic respiration (Basan et al., 2015). Consequently, individual cells typically do not convert sugar completely into SCFAs; instead, they secrete intermediates as a result of overflow metabolism, such as lactate, succinate, and fumarate (Culp and Goodman, 2023). Other microbes then take up these intermediates to complete fermentation, ultimately producing SCFAs. This cross-feeding results in the accumulation of SCFAs at high concentrations in the gut (Culp and Goodman, 2023). In addition to the diet carbohydrates, fermentation of complex carbohydrates from host mucins by gut commensals also contributes to SCFA production (Krautkramer et al., 2021; Ndeh and Gilbert, 2018). In our study, we observed an enrichment of the KEGG pathway “glycosaminoglycan degradation” in individuals with obesity. We also noted an enrichment of the KEGG pathway “glutamate metabolism,” which can be fermented to produce butyrate and propionic acid (Ecklu-Mensah et al., 2023). Collectively, these findings suggest a greater capability for energy harvest through SCFAs, mainly propionic acid, within the microbiome of obese individuals.

Low-grade inflammation is one of the hallmarks of obesity and related metabolic disorders (Portincasa et al., 2023; Mohammad and Thiemermann, 2020; Barathikannan et al., 2019). Increased intestinal permeability, also termed a “leaky gut,” was strongly linked with the origin of this inflammation (Portincasa et al., 2023; DiMattia et al., 2023). In this study, we found that serum LPS, marker of microbial translocation, was positively correlated with BMI and HOMA-IR, consistent with previous reports (Murga-Garrido et al., 2023). Functional metagenomic analysis revealed significant enrichment of several pro-inflammatory KEGG pathway, including “lipopolysaccharide biosynthesis,” “antigen nucleotide sugar biosynthesis,” “biofilm formation Vibrio cholerae,” and “cationic antimicrobial peptide CAMP resistance” in the obese group. Additionally, we observed an enrichment in the pathway “glycosaminoglycan degradation.” Glycosaminoglycan is one of the major components of the intestinal mucus layer (Ndeh and Gilbert, 2018), outer of which is densely colonized by microorganisms (Lee et al., 2022). Excessive degradation of glycosaminoglycans can erode the colonic mucus barrier (Lee et al., 2022). Deficiencies in mucus can disrupt commensal homeostasis and increase susceptibility to pathogens (Yang et al., 2022; McCallum and Tropini, 2024), facilitating the development of obesity via inflammation-related mechanisms. These findings functionally indicate that the microbiota of obese individuals has a greater capacity for growth of potential opportunistic pathogens and favors a pro-inflammatory and pathogenic state.

Consistently, the LEfSe results showed that several pro-inflammatory and pathogenic taxa were significantly enriched in obesity, such as *Proteobacteria* (Portincasa et al., 2023), *Fusobacteri* (Gao et al., 2018), *Enterobacteriaceae* (Jian et al., 2020), *Escherichia* (Cornejo-Pareja et al., 2024), *Morganella* (Yi et al., 2022), and *Paraprevotella* (Riggen-Bueno et al., 2024). Among them, *Proteobacteria* is the primary source of LPS and is often associated with elevated levels of circulating LPS (Ecklu-Mensah et al., 2023; Portincasa et al., 2023). Moreover, *Proteobacteria* often accompanied a high fat/high sugar diet (Ecklu-Mensah et al., 2023), which aligns with the dietary preferences of obese individuals in our study. We also observed that a cluster of *Streptococcus* species (seven species) was enriched in obesity, most of which are potentially pathogenic (Jian et al., 2020; Tan et al., 2022). For example, *Streptococcus oralis* was demonstrated to inhibit inflammasomes, key components of the innate immune system, contributing to the pathogen colonization of the host through release H_2_O_2_ (Erttmann and Gekara, 2019). Given the higher serum LPS observed in obesity, these finding suggest that the overgrowth of potential opportunistic pathogens in the microbiome of obese individuals may contribute to the deterioration of gut barrier integrity, increasing the transport of LPS to circulation. Notably, correlation analysis revealed that fecal thiamine levels were negatively correlated with the majority of opportunistic pathogens enriched in obesity (e.g., *Proteobacteria, Escherichia*, and *Streptococcus oralis*).

Conversely, several potential anti-obesity taxa were observed to be depleted in obesity, such as *Christensenellaceae, Alistipes*, and *Bifidobacterium*. *Christensenellaceae* is emerging as an important player in human health. The relative abundance of *Christensenellaceae* has been reported to be inversely related to obesity and visceral fat (Waters and Ley, 2019). Additionally, it has been shown that the abundance of *Christensenellaceae* increases in response to weight loss by dietary interventions with prebiotic fibers (Riggen-Bueno et al., 2024). *Alistipes* has been reported to likely provide protective effects for individuals with obesity and metabolic diseases (Portincasa et al., 2023). For example, *Alistipes obesi* was significantly enriched in lean individuals, and its increase during dieting was strongly associated with weight loss (Jie et al., 2021). At the species level, we observed that several probiotics was enriched in healthy controls, including *Christensenella sp Marseille* P3954 (Waters and Ley, 2019), *Ruminococcus bicirculans* (Moutsoglou et al., 2023), *Faecalibacterium* spp. (Gao et al., 2018), *Dysosmobacter welbionis* (Portincasa et al., 2023), *Lactobacillus acidophilus* (Portincasa et al., 2023), and multiple *Bifidobacterium species* (Chanda and De, 2024; Chen et al., 2024). Many of them have been reported to improve metabolic disorders, including obesity. For example, *Dysosmobacter welbionis* has been reported to be inversely associated with BMI, and HbA1c in overweight or obese people with metabolic syndrome (Le Roy et al., 2022). Notably, correlation analysis revealed that fecal thiamine levels were positively associated with most of these beneficial taxa. It is worth mentioning that *Dysosmobacter welbionis* is considered to be a strong candidate for the development of next generation beneficial bacteria targeting obesity and associated metabolic diseases (Le Roy et al., 2022). Collectively, these findings suggest that the lower fecal thiamine levels are associated with an imbalanced microbiota in obese individuals.

A major health benefit provided by the gut microbiota is protection against pathogen colonization and subsequent infection, a phenomenon known as colonization resistance (Sorbara and Pamer, 2019; Spragge et al., 2023). Colonization resistance is a collective property of microbiome communities, where individual species cannot effectively resist pathogens (Spragge et al., 2023). Competition for nutrients, including B vitamins, was one of the ways that colonization resistance arises (Caballero-Flores et al., 2023). As microbiome diversity and complexity increases, the likelihood that different nutrients are consumed increases, which helps inhibit pathogen growth and enhance colonization resistance (Spragge et al., 2023). In summary, microbiome diversity and complexity could protect against pathogens by nutrient blocking. Consistent with the observed loss of diversity and complexity in obesity-enriched species, metagenomic functional analysis revealed that VFDB category related to “antimicrobial activity/competitive advantage” is enriched in obese group. These results indicate a compromised colonization resistance in obese microbiome. It has been well recognized that cross-feeding of B vitamins between prototrophic and auxotrophic species strongly contribute to the homeostasis of microbial communities in the distal gut (Rodionov et al., 2019). This mechanism is crucial for shaping community composition, responding to perturbation (Culp and Goodman, 2023). Given the extensive associations between thiamine and microbial taxa discussed above, it is reasonable to speculate that a deficiency of gut thiamine in obesity may disrupt bacteria-bacteria cross-feeding on thiamine. This disruption could further reduce microbial diversity and complexity, ultimately impairing the ability to protect against pathogens through nutrient blocking.

Microbiota-derived vitamins is also involved in the cross-talk between host and microbes. Research indicates that B vitamins play important roles in the maintenance of immune homeostasis (Kunisawa and Kiyono, 2013; Steinert et al., 2020; Yoshii et al., 2019), which is crucial to maintain the symbiotic relationship between host and microbiota (Belkaid and Harrison, 2017). Regarding thiamine, microbiota-derived thiamine could be absorbed by the intestinal epithelium via thiamine transporters (e.g., THTR-1, THTR-2) (Yoshii et al., 2019). Due to its involvement in the intermediary carbon and energy metabolism, thiamine is also involved in immunometabolism in the gut (intestinal integrity and intestinal-linked immune cells), and its deficiency is likely to affect the immune response (Zhan et al., 2022). This aligns with fact that vitamin deficiency can lead to increased susceptibility to infection (Kunisawa and Kiyono, 2013). It was reasonable to speculate that the pro-inflammatory status in obese microbiome might partially due to the impaired gut immunity caused by thiamine deficiency.

In addition to the compromised colonization resistance of gut commensals, the first crucial step for bacterial pathogens to establish a productive infection needs nutrients and proliferation (Cameron and Sperandio, 2015; Mirzaei et al., 2022). Microbiota-derived SCFAs and succinate (a precursor of propionic acid) were important nutrient sources for invading gut pathogens during infection (Cameron and Sperandio, 2015). This aligns with the observed overgrowth of opportunistic pathogens and overproduction of SCFAs, particularly propionic acid, in the gut of individuals with obesity. Furthermore, KEGG pathway “glycosaminoglycan degradation” was observed to be enriched in obesity. The liberated mucosal sugars could directly feed invading pathogen populations (Cameron and Sperandio, 2015). Upon dominated the gut microbiota, bacterial pathogens should control where and when to initiate their virulence process. It has been reported that SCFAs can act as signals for gut opportunistic pathogens that modulate the expression of their virulence traits and eventually influence the trigger of infection (Cameron and Sperandio, 2015; Mirzaei et al., 2022). Notably, total SCFAs was observed to be positively correlated with serum LPS, suggesting that excess SCFAs might contribute to the microbial translocation in obesity. Research indicates that due to the excess and intestinal dissemination of SCFAs, bacterial pathogens may utilize these SCFAs as biogeographical signals to discriminate among various locations in the intestine. Among pathogenic bacteria, *Escherichia coli* is the well-studied pathogens could sense and use SCFAs for growth and virulence, consequently stimulating inflammation during dysbiosis. Notably, *Escherichia coli* was observed to be remarkably enriched in obese group. It has been noted that *Escherichia coli* could sense and use SCFAs, mainly to the regulation of virulence gene expression (Mirzaei et al., 2022). For example, one study showed that propionic acid could induce the expression of points and transcription factors that cause macrophage susceptibility to colonization by enteropathogenic bacteria (Pobeguts et al., 2020). Another study showed that exposure to propionic acid could result in adherent-invasive *Escherichia coli* (AIEC) resistance and enhanced virulence in its occupancy (Ormsby et al., 2020). These findings suggest that excess SCFAs, particularly propionic acid, might be an important contributor to the development of obesity via inflammation-related mechanisms in addition to contributing to the energy harvest from gut.

It is important to note that there are some limitations in our study. Sample size of the study was relatively small, an in-depth stratified analyses of obesity sub-phenotypes is warranted to validate our findings in a larger population; due to the observational nature of the present research, we primarily focused on biologically plausible mechanisms based on correlations between fecal thiamine, microbiota, and host phenotypes. Future research should investigate the dynamic changes of gut microbial thiamine metabolism before and after weight loss interventions through prospective clinical trials. It is also essential to validate the causal relationship between gut thiamine levels and obesity-related phenotypes through high-fat diet-induced obese rodent model.

## 5 Conclusion

To the best of our knowledge, this exploratory study is the first human study to examine the relationship between gut B vitamins, gut microbiota, and obesity-related phenotypes. Our findings indicate that low fecal thiamine levels in obese individuals are associated with impaired thiamine biosynthesis by gut microbiota. The broad associations between fecal thiamine, the gut microbiota, and obesity-related phenotypes suggest that targeting gut microbial thiamine metabolism could be a promising therapeutic approach for obesity. Future research should focus on establishing causal relationships between improved obesity outcomes and the regulation of gut thiamine metabolism in animal experiments and clinical studies.

## Data Availability

The original contributions presented in the study are publicly available. This data can be found here: https://www.ncbi.nlm.nih.gov, accession number PRJNA1110638.
