# Supplementary material for: Multi-omics analyses reveal altered gut microbial thiamine production in obesity
Source: Front Microbiol. 2025 Jun 17;16:1516393. doi: 10.3389/fmicb.2025.1516393 (PMC12209360; doi:10.3389/fmicb.2025.1516393)
Supplement: Supplementary file 2 [file Data_Sheet_1.pdf]

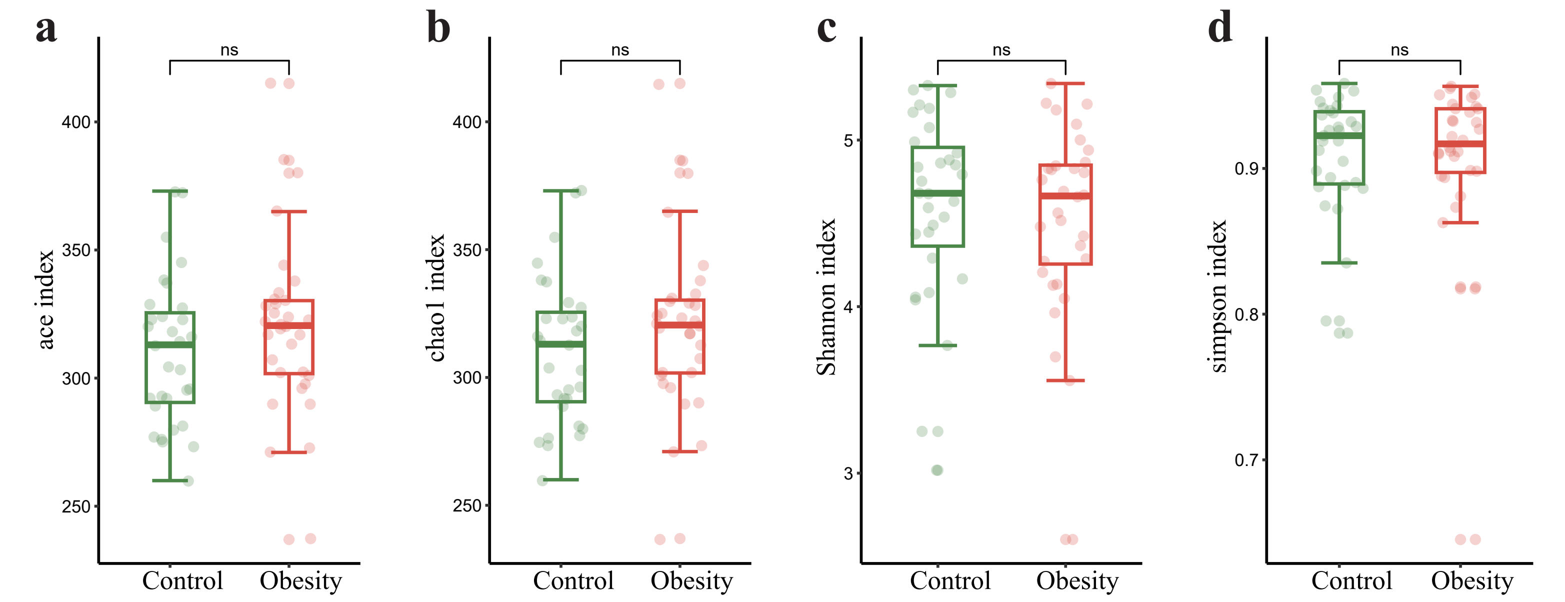

Figure S1 Box plot of bacterial alpha diversity the in control and obese individuals. (a-d) Bacterial alpha diversity metrics measured by the Simpson, chao1, ace, and Shannon indexes, respectively. ns represents no significant.

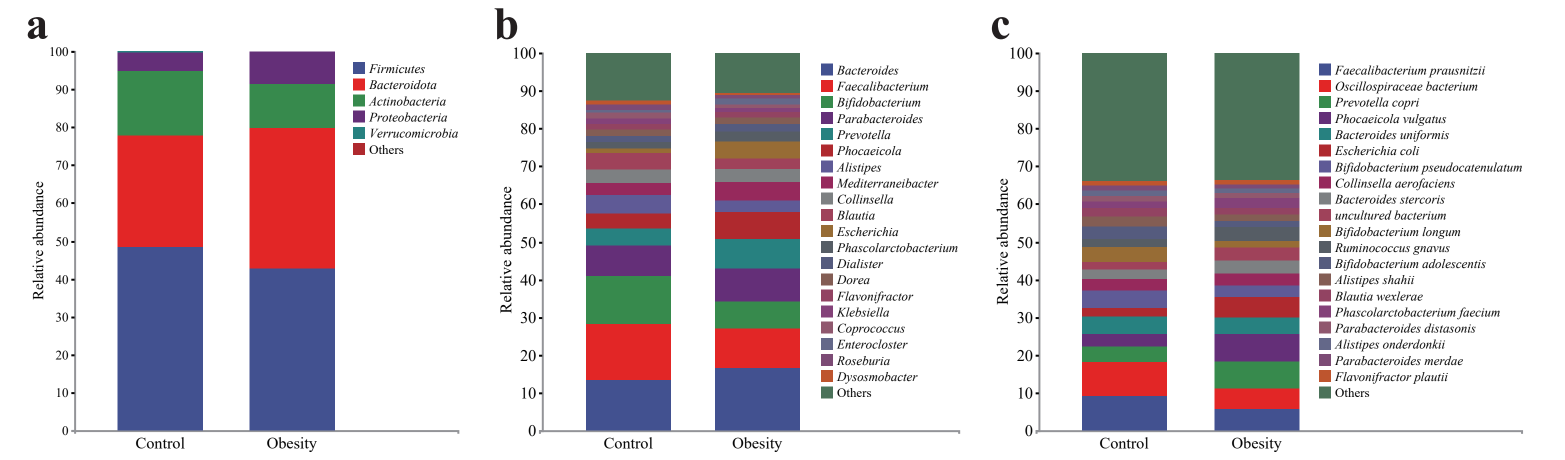

Figure S2 Distribution of microbial taxa in two groups. (a-c) Distribution of bacteria at the phylum-level, genus-level, and species-level in the fecal microbiota of obese subjects and healthy control subjects, respectively.

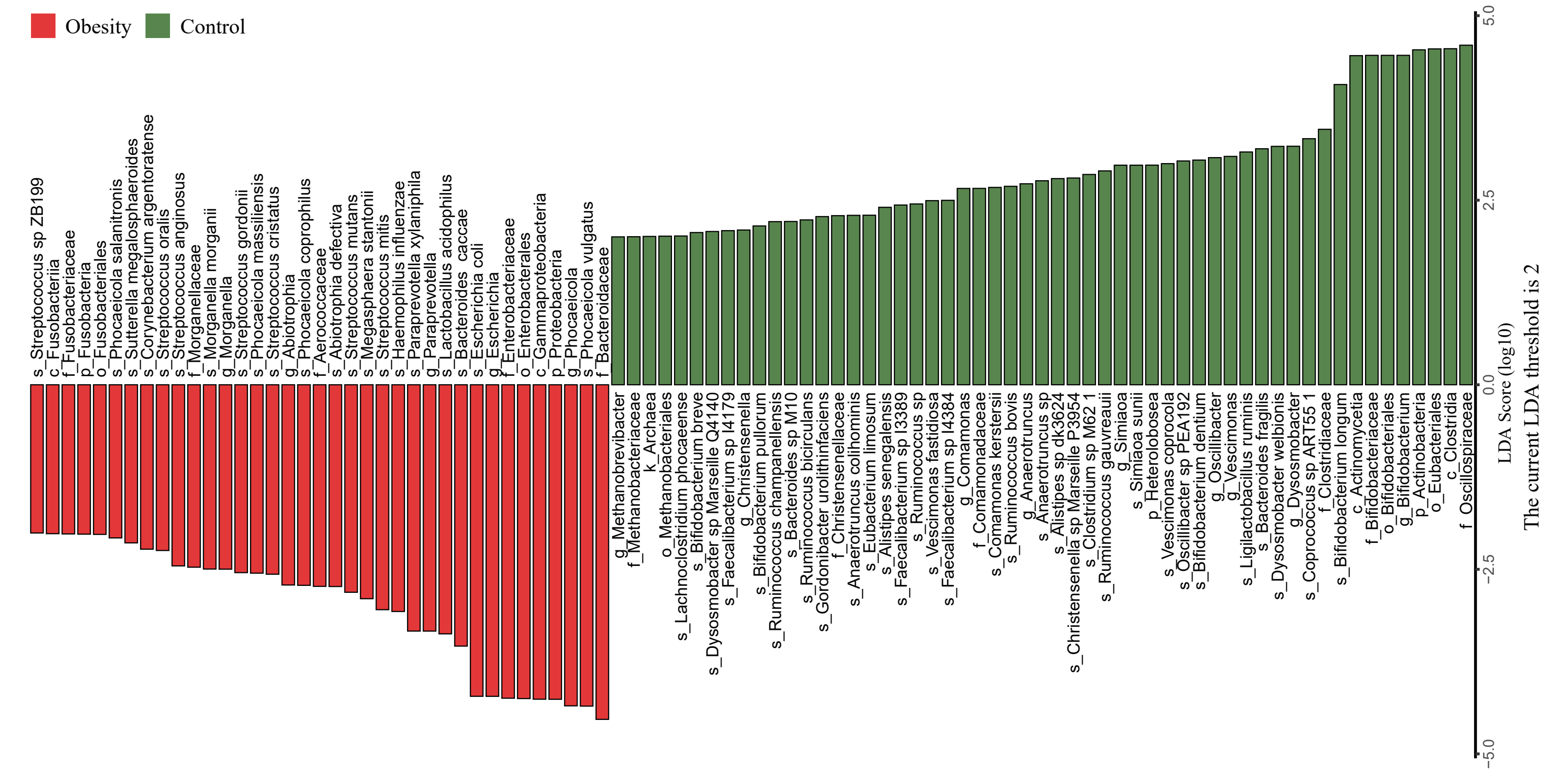

Figure S3 Taxa identified by LEfSe as biomarkers of samples from the control or obese groups (cutoffs were LDA score [log 10] > 2). Red bars indicate taxa were enrichment in obesity, and green bars indicate taxa were enrichment in healthy controls.

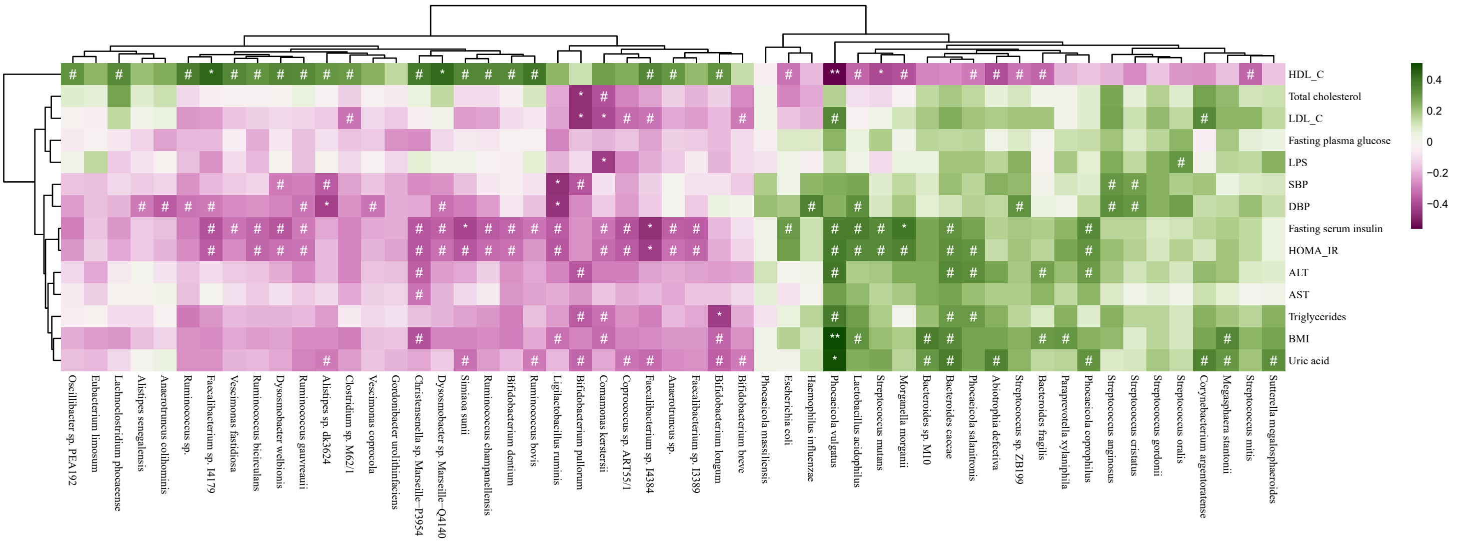

Figure S4 Associations of gut microbial species with obesity-related phenotypes. Heat map of the Spearman's rank correlation coefficient between 14 clinical indices and 53 BMI-associated species. Green color represents a positive correlation while purple color represents a negative correlation. HDL-C, high-density lipoprotein cholesterol; LDL-C, low-density lipoprotein cholesterol; LPS, lipopolysaccharide; SBP, systolic blood pressure; DBP, diastolic blood pressure; HOMA-IR, insulin resistance index; ALT, alanine transaminase; AST, aspartate transaminase; BMI, body mass index. $^{\#}P_{FDR} < 0.1$; $^{*}P_{FDR} < 0.05$; $^{**}P_{FDR} < 0.01$.

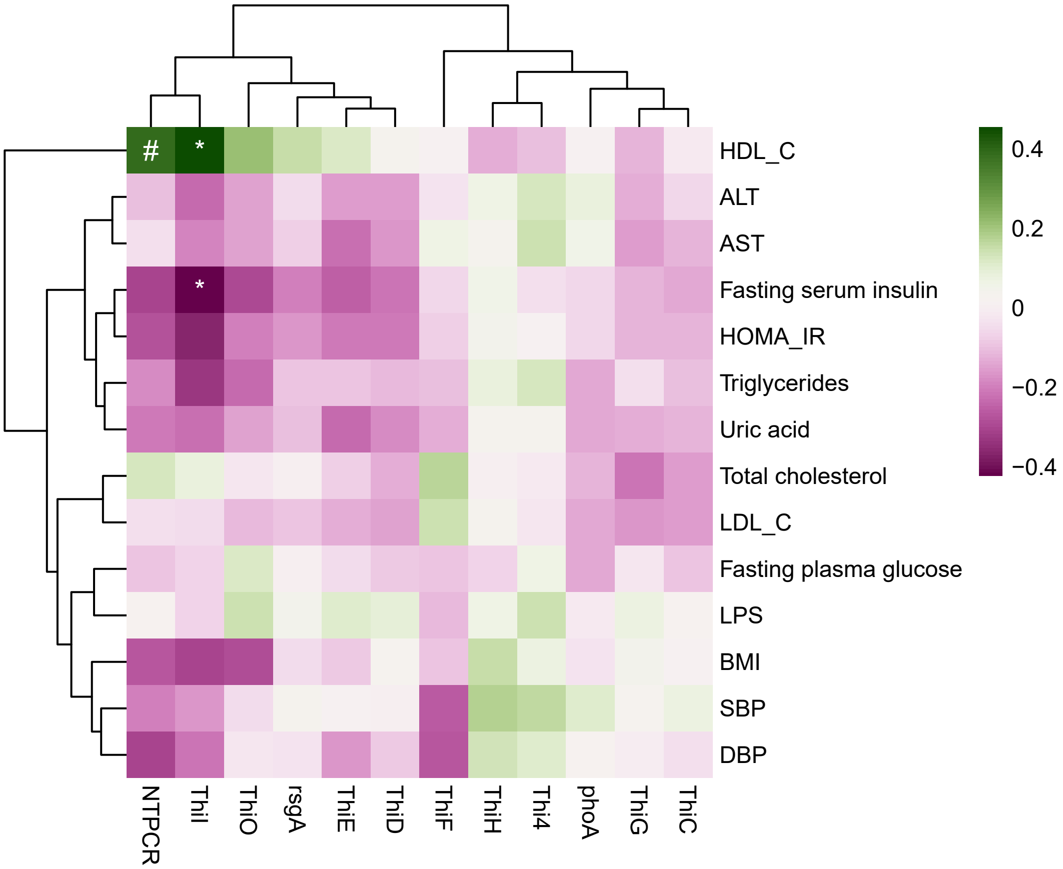

Figure S5 Associations of enzymes involved in the microbial production of thiamine and obesity-related phenotypes. Heat map of the Spearman's rank correlation coefficient between 12 enzymes and 14 clinical indices. Green color represents a positive correlation while purple color represents a negative correlation. $^{\#}P_{FDR} < 0.1$; $^{*}P_{FDR} < 0.05$.
